# Supplementary material for: Mental health of individuals infected with SARS-CoV-2 during mandated isolation and compliance with recommendations—A population-based cohort study
Source: PLoS One. 2022 Mar 16;17(3):e0264655. doi: 10.1371/journal.pone.0264655 (PMC8926272; doi:10.1371/journal.pone.0264655)
Supplement: S1 File — (DOCX) [file pone.0264655.s001.docx]

Supplementary Material

[Table S1 2](#_Toc94606819)

[Table S2 3](#_Toc94606820)

[Tables S3a – S3i 4](#_Toc94606821)

[Table S4 7](#_Toc94606822)

[Tables S5a and S5b 8](#_Toc94606823)

[Table S6 10](#_Toc94606824)

[Tables S7a – S7c 11](#_Toc94606825)

[Figure S1a 12](#_Toc94606826)

[Figure S1b 13](#_Toc94606827)

[Figure S2 14](#_Toc94606828)

[Figures S3a – S3c 15](#_Toc94606829)

[Figures 4a – 4c 16](#_Toc94606830)

[Figures S5a – S5c 17](#_Toc94606831)

# Table S1

| **Table S1: Characteristics of Participants enrolled in the Zurich SARS-CoV-2 Cohort study presented according to living situation** | | | |
| --- | --- | --- | --- |
|  | **Living alone** | **At least 1 other household member** | |
|  |  | **Including Children** | |
|  |  | **Yes** | **No** |
|  | (N=229) | (N=369) | (N=949) |
| **Sex** |  |  |  |
| Female | 139 (60.7%) | 189 (51.2%) | 455 (47.9%) |
| Male | 90 (39.3%) | 180 (48.8%) | 494 (52.1%) |
| **Age Group** |  |  |  |
| 18-39 years | 65 (28.4%) | 125 (33.9%) | 323 (34.0%) |
| 40-64 years | 86 (37.6%) | 232 (62.9%) | 339 (35.7%) |
| 65+ years | 78 (34.1%) | 12 (3.3%) | 287 (30.2%) |
| **Age (years)** |  |  |  |
| Median [Min, Max] | 55.0 [18.0, 88.0] | 43.0 [17.0, 79.0] | 54.0 [18.0, 92.0] |
| Median [IQR] | 55.0 [37.0, 68.0] | 43.0 [37.0, 49.0] | 54.0 [32.0, 66.0] |
| **Symptom Severity of COVID-19** |  |  |  |
| Asymptomatic | 20 (8.7%) | 42 (11.4%) | 133 (14.0%) |
| Mild | 38 (16.6%) | 85 (23.0%) | 194 (20.4%) |
| Moderate | 93 (40.6%) | 156 (42.2%) | 378 (39.8%) |
| Severe | 51 (22.3%) | 65 (17.6%) | 188 (19.8%) |
| Very Severe | 11 (4.8%) | 21 (5.7%) | 53 (5.6%) |
| Missing | 16 (7%) | 0 | 3 (0.3%) |
| **Comorbidities** |  |  |  |
| One or more comorbidities | 85 (37.1%) | 82 (22.2%) | 305 (32.1%) |
| Missing | 17 (7.4%) | 0 (0%) | 0 (0%) |
| **Number of Days in Isolation*** |  |  |  |
| Mean (SD) | 11.4 (2.28) | 10.8 (2.46) | 11.0 (2.33) |
| Median [Min, Max] | 10.0 [5.00, 20.0] | 10.0 [2.00, 23.0] | 10.0 [4.00, 25.0] |
| Missing | 131 (57.2%) | 172 (46.6%) | 415 (43.7%) |
| **Education** |  |  |  |
| None or mandatory school | 5 (2.2%) | 19 (5.1%) | 42 (4.4%) |
| Federal Diploma | 91 (39.7%) | 134 (36.2%) | 422 (44.5%) |
| Higher technical school or college | 61 (26.6%) | 89 (24.1%) | 245 (25.8%) |
| University | 47 (20.5%) | 126 (34.1%) | 239 (25.2%) |
| Missing | 25 (10.9%) | 1 (0.3%) | 1 (0.1%) |
| **Job** |  |  |  |
| Employed | 108 (47.2%) | 265 (71.8%) | 523 (55.1%) |
| Self Employed | 14 (6.1%) | 45 (12.2%) | 89 (9.4%) |
| Student | 5 (2.2%) | 17 (4.6%) | 50 (5.3%) |
| Retired | 70 (30.6%) | 11 (3.0%) | 258 (27.1%) |
| Unemployed | 10 (4.4%) | 16 (4.3%) | 21 (2.2%) |
| Family manager | 0 (0%) | 12 (3.3%) | 6 (0.6%) |
| Missing | 22 (9.6%) | 3 (0.8%) | 2 (0.2%) |
| **Monthly household income (Swiss Francs)** |  |  |  |
| <6'000 | 87 (38.0%) | 87 (23.6%) | 318 (33.3%) |
| 6'000 - 12'0000 | 95 (41.5%) | 136 (36.9%) | 383 (40.4%) |
| >12'000 | 16 (7.0%) | 129 (35.0%) | 201 (21.2%) |
| Missing | 31 (13.5%) | 17 (4.6%) | 49 (5.2%) |
| **Cohort** |  |  |  |
| Prospectively recruited | 161 (70.3%) | 251 (68.0%) | 693 (73.0%) |
| Retrospectively recruited | 68 (29.7%) | 118 (32.0%) | 256 (27.0%) |

*Only assessed in the prospective cohort

# Table S2

| **Table S2: Multivariable ordinal regression evaluating the influence of sociodemographic background on Information Status** | | | |
| --- | --- | --- | --- |
| Total Cohort | | | |
|  | **Odds Ratios** | **95% CI** | **p-value** |
| Sex: male | 1.07 | 0.88 – 1.30 | 0.505 |
| Living with Children | 1.42 | 1.12 – 1.79 | **0.004** |
|  |  |  |  |
| **Age group** | | | |
| **Reference: 18 – 39 years old** |  |  |  |
| Age: 40 – 64 years old | 0.65 | 0.51 – 0.82 | **<0.001** |
| Age: 65+ years old | 1.12 | 0.68 – 1.86 | 0.656 |
|  |  |  |  |
| **Education** | | | |
| **Reference: none or mandatory** |  |  |  |
| Vocational training and specialized baccalaureate | 1.07 | 0.66 – 1.74 | 0.777 |
| Higher technical school or college | 0.84 | 0.51 – 1.39 | 0.489 |
| University | 0.78 | 0.47 – 1.29 | 0.332 |
|  |  |  |  |
| **Occupation** | | | |
| **Reference: employed** |  | | |
| Self-employed | 1.27 | 0.90 – 1.80 | 0.179 |
| In Education | 0.81 | 0.50 – 1.31 | 0.388 |
| Retired | 0.81 | 0.49 – 1.34 | 0.419 |
| Without Work | 0.91 | 0.51 – 1.61 | 0.738 |
| Family Manager | 2.11 | 0.89 – 4.98 | 0.088 |
|  |  |  |  |
| Observations* | 1510/1547 |  |  |
| R2 Nagelkerke | 0.055 |  |  |

* Only analyzed in Participants who stated information status

Table S2 describes association of sociodemographic background with how well participants felt they were briefed on specific isolation recommendations in all 1510 participants who stated their information level.

# Tables S3a – S3i

| **Table S3a: Percentage of Participants with Depression in DASS-21 Categories at Baseline and 2 Weeks after diagnosis** | | | | | | | | |
| --- | --- | --- | --- | --- | --- | --- | --- | --- |
| Prospectively recruited Cohort | | | | | | | | |
|  | **Week 2** | | | | | | | |
| **Baseline** | **No depression** | **Mild depression** | **Moderate depression** | **Severe depression** | | **Extremely severe depression** | **Missing** | **Total** |
| **No depression** | 743(95.9%) | 53(74.6%) | 33(52.4%) | 6(46.2%) | | 5(38.5%) | 119(82.6%) | 959(88.9%) |
| **Mild depression** | 23(2.8%) | 13(18.3%) | 5(7.9%) | 1(7.7%) | | 2(15.4%) | 8(5.6%) | 51(4.7%) |
| **Moderate depression** | 7(0.9%) | 4(5.6%) | 18(28.6%) | 4(30.8%) | | 4(30.8%) | 2(1.4%) | 39(3.6%) |
| **Severe depression** | 1(0.1%) | 0 | 6(9.5%) | 1(7.7%) | | 0 | 1(0.7%) | 9(0.8%) |
| **Extremely severe depression** | 1(0.1%) | 1(1.4%) | 1(1.6%) | 1(7.7%) | | 2(15.4%) | 2(1.4%) | 8(0.7%) |
| **Missing** | 1(0.1%) | 0 | 0 | 0 | | 0 | 12(8.3%) | 13(1.2%) |
| **Total*** | 775(100%) | 71(100%) | 63(100%) | 13(100%) | | 13(100%) | 144(100%) | 1079(100%) |
| **Table S3b: Percentage of Participants with Depression in DASS-21 Categories 2 Weeks and 1 Month after diagnosis** | | | | | | | | |
| Prospectively recruited Cohort | | | | | | | | |
|  | **Month 1** | | | | | | | |
| **Week 2** | **No depression** | **Mild depression** | **Moderate depression** | | **Severe depression** | **Extremely severe depression** | **Missing** | **Total** |
| **No depression** | 721(82.9%) | 22(34.9%) | 17(28.3%) | | 1(6.2%) | 2(18.2%) | 12(20.3%) | 775 (71.8%) |
| **Mild depression** | 43(4.9%) | 16(25.4%) | 9(15.0%) | | 2(12.5%) | 0 | 1(1.7%) | 71(6.6%) |
| **Moderate depression** | 16(1.8%) | 12(19.0%) | 22(36.7%) | | 8(50%) | 5(45.5%) | 0 | 63(5.8%) |
| **Severe depression** | 4(0.5%) | 4(6.3%) | 2(3.3%) | | 2(12.5%) | 1(9.1%) | 0 | 13(1.2%) |
| **Extremely severe depression** | 1(0.1%) | 1(1.6%) | 7(11.7%) | | 1(6.2%) | 3(27.3%) | 0 | 13(1.2%) |
| **Missing** | 85(9.8%) | 8(12.7%) | 3(5.0%) | | 2(12.5%) | 0 | 46(78.0%) | 144(13.3%) |
| **Total*** | 870(100%) | 63(100%) | 60(100%) | | 16(100%) | 11(100%) | 59(100%) | 1079(100%) |
| **Table S3c: Percentage of Participants with Depression in DASS-21 Categories at Baseline and 1 Month after diagnosis** | | | | | | | | |
| Prospectively recruited Cohort | | | | | | | | |
|  | **Month 1** | | | | | | | |
| **Baseline** | **No depression** | **Mild depression** | **Moderate depression** | | **Severe depression** | **Extremely severe depression** | **Missing** | **Total** |
| **No depression** | 831(95.5%) | 42 (66.7%) | 34(56.7%) | | 5(31.2%) | 5(45.5%) | 42(71.2%) | 959 (88.9%) |
| **Mild depression** | 27(3.1%) | 11(17.5%) | 8(13.3%) | | 3(18.8%) | 0 | 2(3.4%) | 51(4.7%) |
| **Moderate depression** | 9(1.0%) | 8(12.7%) | 14(23.3%) | | 5(31.2%) | 1(9.1%) | 2(3.4%) | 39(3.6%) |
| **Severe depression** | 0 | 2(3.2%) | 3(5.0%) | | 2(12.5%) | 2(18.2%) | 0 | 9(0.8%) |
| **Extremely severe depression** | 1(0.1%) | 0 | 1(1.7%) | | 1(6.2%) | 3(27.3%) | 2(3.4%) | 8(0.7%) |
| **Missing** | 2(0.2%) | 0 | 0 | | 0 | 0 | 11(18.6%) | 13(1.2%) |
| **Total*** | 870(100%) | 63(100%) | 60(100%) | | 16(100%) | 11(100%) | 59(100%) | 1079(100%) |

| **Table S3d: Percentage of Participants with Anxiety in DASS-21 Categories at Baseline and 2 Weeks after diagnosis** | | | | | | | | | | | |
| --- | --- | --- | --- | --- | --- | --- | --- | --- | --- | --- | --- |
| Prospectively recruited Cohort | | | | | | | | | | | |
|  | | **Week 2** | | | | | | | | | |
| **Baseline** | | **No anxiety** | | **Mild anxiety** | | **Moderate anxiety** | **Severe anxiety** | | **Extremely severe anxiety** | **Missing** | **Total** |
| **No anxiety** | | 741(96.4%) | | 48(76.2%) | | 49(73.1%) | 6(37.5%) | | 5(27.8%) | 120(82.2%) | 969(89.8%) |
| **Mild anxiety** | | 9(1.2%) | | 8(21.7%) | | 5(7.5%) | 6(37.5%) | | 1(5.6%) | 6(4.1%) | 35(3.2%) |
| **Moderate anxiety** | | 15(2.0%) | | 7(11.1%) | | 9(13.4%) | 1(6.2%) | | 4(22.2%) | 7(4.8%) | 43(4.0%) |
| **Severe anxiety** | | 2(0.3%) | | 0 | | 2(3.0%) | 1(6.2%) | | 3(16.7%) | 0 | 8(0.7%) |
| **Extremely severe anxiety** | | 1(0.1%) | | 0 | | 2(3.0%) | 2(12.5%) | | 5(27.8%) | 1(0.7%) | 11(1.0%) |
| **Missing** | | 1(0.1%) | | 0 | | 0 | 0 | | 0 | 12(8.2%) | 13(1.2%) |
| **Total** | | 769(100%) | | 63(100%) | | 67(100%) | 16(100%) | | 18(100%) | 146(100%) | 1079(100.0%) |
| **Table S3e: Percentage of Participants with Anxiety in DASS-21 Categories at 2 Weeks and 1 Month after diagnosis** | | | | | | | | | | | |
| Prospectively recruited Cohort | | | | | | | | | | | |
|  | | **Month 1** | | | | | | | | | |
| **Week 2** | | **No anxiety** | | **Mild anxiety** | | **Moderate anxiety** | **Severe anxiety** | | **Extremely severe anxiety** | **Missing** | **Total** |
| **No anxiety** | | 717(82.6%) | | 17(34.7%) | | 19(31.1%) | 3(15%) | | 1(4.8%) | 12(20%) | 769(71.3%) |
| **Mild anxiety** | | 43(5.0%) | | 12(24.5%) | | 7(11.5%) | 0 | | 0 | 1(1.7%) | 63(5.8%) |
| **Moderate anxiety** | | 23(2.6%) | | 13(26.5%) | | 19(31.1%) | 7(35%) | | 5(23.8%) | 0 | 67(6.2%) |
| **Severe anxiety** | | 0 | | 1(2.0%) | | 6(9.8%) | 4(20%) | | 5(23.8%) | 0 | 16(1.5%) |
| **Extremely severe anxiety** | | 2(0.2%) | | 0 | | 2(3.3%) | 5(25%) | | 8(38.1%) | 1(1.7%) | 18(1.7%) |
| **Missing** | | 83(9.6%) | | 6(12.2) | | 8(13.1%) | 1(5.0%) | | 2(9.5%) | 46(76.7%) | 146(13.5%) |
| **Total** | | 868(100%) | | 49(100%) | | 61(100%) | 20(100%) | | 21(100%) | 60(100%) | 1079(100%) |
| **Table S3f: Percentage of Participants with Anxiety in DASS-21 Categories at Baseline and 1 Month after diagnosis** | | | | | | | | | | | |
| Prospectively recruited Cohort | | | | | | | | | | | |
|  | **Month 1** | | | | | | | | | | |
| **Baseline** | **No anxiety** | | **Mild anxiety** | | **Moderate anxiety** | | | **Severe anxiety** | **Extremely severe anxiety** | **Missing** | **Total** |
| **No anxiety** | 829(95.5%) | | 43(87.8%) | | 39(63.9%) | | | 6(30%) | 10(47.6%) | 42(70%) | 969(89.8%) |
| **Mild anxiety** | 16(1.8%) | | 3(6.1%) | | 7(11.5%) | | | 4(20.0%) | 3(14.3% | 2(3.3%) | 35(3.2%) |
| **Moderate anxiety** | 18(2.1%) | | 3(6.1%) | | 13(21.3%) | | | 4(20.0%) | 2(9.5% | 3(5.0%) | 43(4.0%) |
| **Severe anxiety** | 2(0.2%) | | 0 | | 1(1.6%) | | | 3(15.0%) | 2(9.5% | 0 | 8(0.7%) |
| **Extremely severe anxiety** | 1(0.1%) | | 0 | | 1(1.6%) | | | 3(15.0%) | 4(19%) | 2(3.3%) | 11(1.0%) |
| **Missing** | 2(0.2%) | | 0 | | 0 | | | 0 | 0 | 11(18.3%) | 13(1.2%) |
| **Total** | 868(100%) | | 49(100%) | | 61(100%) | | | 20(100%) | 21(100%) | 60(100%) | 1079(100%) |

| **Table S3g: Percentage of Participants with Stress in DASS-21 Categories at Baseline and 2 Weeks after diagnosis** | | | | | | | | | | | | | |
| --- | --- | --- | --- | --- | --- | --- | --- | --- | --- | --- | --- | --- | --- |
| Prospectively recruited Cohort | | | | | | | | | | | | | |
|  | **Week 2** | | | | | | | | | | | | |
| **Baseline** | **No stress** | | **Mild stress** | **Moderate stress** | **Severe stress** | | | **Extremely severe stress** | **Missing** | | **Total** | | |
| **No stress** | 818(95.9%) | | 25(65.8%) | 18(60.0%) | 45.5%) | | | 0 | 123(85.4%) | | 989(91.7%) | | |
| **Mild stress** | 18(2.1%) | | 7(18.4%) | 6(20.0%) | 1(9.1%) | | | 0 | 0 | | 32(3.0%) | | |
| **Moderate stress** | 14(1.6%) | | 3(7.9%) | 3(10.0%) | 3(27.3%) | | | 3(100%) | 6(4.2%) | | 32(3.0%) | | |
| **Severe stress** | 1(0.1%) | | 2(5.3%) | 3(10.0%) | 1(9.1%) | | | 0 | 2(1.4%) | | 9(0.8%) | | |
| **Extremely severe stress** | 1(0.1%) | | 0 | 0 | 1(9.1%) | | | 0 | 1(0.7%) | | 3(0.3%) | | |
| **Missing** | 1(0.1%) | | 1(2.6%) | 0 | 0 | | | 0 | 812(.3%) | | 14(1.3%) | | |
| **Total** | 853(100%) | | 38(100%) | 30(100%) | 11(100%) | | | 3(100%) | 144(100%) | | 1079(100%) | | |
| **Table S3h: Percentage of Participants with Stress in DASS-21 Categories at 2 Weeks and 1 Month after diagnosis** | | | | | | | | | | | | | |
| Prospectively recruited Cohort | | | | | | | | | | | | | |
|  | **Month 1** | | | | | | | | | | | | |
| **Week 2** | **No stress** | | **Mild stress** | **Moderate stress** | | **Severe stress** | | **Extremely severe stress** | | **Missing** | | **Total** | |
| **No stress** | 802(86.7%) | | 20(54.1%) | 9(28.1%) | | 4(23.5%) | | 1(33.3%) | | 17(26.2%) | | 853(79.1%) | |
| **Mild stress** | 21(2.3%) | | 6816.2%) | 9828.1%) | | 1(5.9%) | | 0 | | 1(1.5%) | | 38(3.5%) | |
| **Moderate stress** | 10(1.1%) | | 4(10.8%) | 8(25.0%) | | 8(47.1%) | | 0 | | 0 | | 30(2.8%) | |
| **Severe stress** | 4(0.4%) | | 0 | 4(12.5%) | | 2(11.8%) | | 1(33.3%) | | 0 | | 1(1.0%) | |
| **Extremely severe stress** | 0 | | 1(2.7%) | 0 | | 1(5.9%) | | 1(33.3%) | | 0 | | 380.3%) | |
| **Missing** | 88(9.5%) | | 6(16.2%) | 2(6.2%) | | 1(5.9%) | | 0 | | 47(72.3%) | | 144(13.3%) | |
| **Total** | 925(100%) | | 37(100%) | 32(100%) | | 17(100%) | | 3(100%) | | 65(100%) | | 1079(100%) | |
| **Table S3i: Percentage of Participants with Stress in DASS-21 Categories at Baseline and 1 Month after diagnosis** | | | | | | | | | | | | |  |
| Prospectively recruited Cohort | | | | | | | | | | | | |  |
|  | | **Month 1** | | | | | | | | | | |  |
| **Baseline** | | **No stress** | **Mild stress** | **Moderate stress** | | | **Severe stress** | **Extremely severe stress** | **Missing** | | **Total** | |  |
| **No stress** | | 887(95.9% | 25(67.6%) | 19(59.4%) | | | 10(58.8%) | 1(33.3%) | 47(72.3%) | | 989(91.7%) | |  |
| **Mild stress** | | 20(2.2%) | 5(13.5%) | 5(15.6%) | | | 1(5.9%) | 1(33.3%) | 0 | | 32(3.0%) | |  |
| **Moderate stress** | | 11(1.2%) | 4(10.8%) | 6(18.8%) | | | 5(29.4%) | 1(33.3%) | 5(7.7%) | | 32(3.0%) | |  |
| **Severe stress** | | 3(0.3%) | 2(5.4%) | 2(6.2%) | | | 1(5.9%) | 0 | 1(1.5%) | | 9(0.8%) | |  |
| **Extremely severe stress** | | 2(0.2%) | 0 | 0 | | | 0 | 0 | 1(1.5%) | | 3(0.3%) | |  |
| **Missing** | | 2(0.2%) | 1(2.7%) | 0 | | | 0 | 0 | 11(16.9%) | | 14(1.3%) | |  |
| **Total** | | 925(100%) | 37(100%) | 32(100%) | | | 17(100%) | 3(100%) | 65(100%) | | 1079(100%) | |  |

**Tables S3a – S3i.** Crosstabulations of Participants classified with different severity levels of Depression, Anxiety and Stress before, 2 Weeks and 1 Month after receiving a positive test result for SARS-CoV-2

# Table S4

| **Table S4: Difficulty of adhering to recommendations form the FOPH** | | | | | | |
| --- | --- | --- | --- | --- | --- | --- |
| Total Cohort | | | | | | |
| N = 1547 | | | | | | |
| **Specific Recommendations** | **Perceived Level of Difficulty** | | | | | |
|  | **Very difficult** | **Difficult** | **Neither** | **Easy** | **Very easy** | **Missing** |
| **Follow recommendations in general** | 63 (4.1%) | 231 (15%) | 389 (26%) | 507 (33%) | 329 (22%) | 28 |
|  | | | | | | |
| Avoid any contact with pets* | 113 (37%) | 88 (29%) | 26 (8.4%) | 32 (10%) | 49 (16%) | 62 |
| Stay alone in a room** | 189 (19%) | 203 (20%) | 196 (19%) | 233 (23%) | 200 (20%) | 297 |
| Clean surfaces daily | 112 (9.5%) | 175 (15%) | 280 (24%) | 343 (29%) | 263 (22%) | 374 |
| Have other people wear mask** | 96 (10%) | 83 (8.6%) | 152 (16%) | 268 (28%) | 365 (38%) | 354 |
| Use a separate bathroom** | 91 (8.6%) | 119 (10%) | 174 (16%) | 315 (30%) | 375 (35%) | 254 |
| Wear a Mask when leaving the room** | 80 (7.8%) | 95 (9.2%) | 154 (15%) | 285 (28%) | 414 (40%) | 290 |
| Not sharing cutlery/dishes** | 79 (7.5%) | 93 (8.9%) | 160 (15%) | 331 (32%) | 387 (37%) | 268 |
| Not sharing laundry** | 80 (7.4%) | 121 (11%) | 184 (17%) | 355 (33%) | 336 (31%) | 242 |
| Get Groceries or Supplies delivered | 12 (0.8%) | 44 (3.1%) | 139 (9.8%) | 476 (34%) | 742 (53%) | 134 |

Percentages applied only to non-missing data, *only analyzed in Participants owning a pet, n =370 **only analyzed in Participants not living alone, n = 1318

# Tables S5a and S5b

| **Table S5a Influence of sociodemographic background on perceived difficulty of Isolation in multivariable ordinal regression** | | | |
| --- | --- | --- | --- |
| Prospective cohort | | | |
|  | **Odds Ratios** | **95% CI** | **p-value** |
| Male Sex | 0.74 | 0.59 – 0.92 | 0.007 |
| Living Alone | 0.52 | 0.38 – 0.73 | <0.001 |
| Living with Children | 2.29 | 1.41 – 3.73 | 0.001 |
| Living with Pets | 1.20 | 0.92 – 1.55 | 0.180 |
|  | | | |
| **Age group** | | | |
| **Reference: 18 – 39 years old** |  |  |  |
| Age: 40 – 64 years old | 0.71 | 0.51 – 0.99 | 0.042 |
| Age: 65+ years old | 0.61 | 0.34 – 1.11 | 0.104 |
|  |  |  |  |
| **Information Status** | | | |
| **Reference: Well informed** |  |  |  |
| Neither poorly nor well informed | 1.52 | 1.05 – 2.21 | 0.028 |
| Poorly informed | 2.18 | 1.16 – 4.08 | 0.015 |
|  |  |  |  |
| **Occupation** |  |  |  |
| **Reference: employed** |  |  |  |
| Self-employed | 1.10 | 0.73 – 1.66 | 0.641 |
| In Education | 0.84 | 0.50 – 1.39 | 0.489 |
| Retired | 0.94 | 0.53 – 1.67 | 0.840 |
| Without Work | 0.86 | 0.44 – 1.68 | 0.660 |
| Family Manager | 0.91 | 0.34 – 2.45 | 0.845 |
|  |  |  |  |
| **Interaction with age (ref: 18-39) when living with children** |  |  |  |
| 40-64 years old | 0.70 | 0.39 – 1.28 | 0.251 |
| 65+ years old | 0.41 | 0.12 – 1.40 | 0.156 |
|  |  |  |  |
| Observations* | 1078/1105 |  |  |
| R2 Nagelkerke | 0.125 |  |  |

* Only analyzed in Participants who stated overall difficulty

**Table S5a** describes association of sociodemographic background with the perceived difficulty level of adherence to isolation recommendations overall in all 1078 participants who stated their overall difficulty level in the prospective cohort.

| **Table S5b Influence of sociodemographic background on perceived difficulty of Isolation in multivariable ordinal regression** | | | |
| --- | --- | --- | --- |
| Retrospective cohort | | | |
|  | **Odds Ratios** | **95% CI** | **p-value** |
| Male Sex | 0.73 | 0.51 – 1.05 | 0.093 |
| Living Alone | 1.43 | 0.83 – 2.47 | 0.200 |
| Living with Children | 2.00 | 1.06 – 3.79 | 0.034 |
| Living with Pets | 0.97 | 0.63 – 1.49 | 0.900 |
|  | | | |
| **Age group** | | | |
| **Reference: 18 – 39 years old** |  |  |  |
| Age: 40 – 64 years old | 1.21 | 0.75 – 1.95 | 0.438 |
| Age: 65+ years old | 1.03 | 0.32 – 3.22 | 0.963 |
|  |  |  |  |
| **Information Status** | | | |
| **Reference: Well informed** |  |  |  |
| Neither poorly nor well informed | 2.47 | 1.52 – 4.04 | <0.001 |
| Poorly informed | 5.52 | 2.70 – 11.39 | <0.001 |
|  |  |  |  |
| **Occupation** |  |  |  |
| **Reference: employed** |  |  |  |
| Self-employed | 0.69 | 0.36 – 1.34 | 0.277 |
| In Education | 1.43 | 0.54 – 3.78 | 0.467 |
| Retired | 0.65 | 0.22 – 1.88 | 0.421 |
| Without Work | 1.08 | 0.47 – 2.49 | 0.185 |
| Family Manager | 3.49 | 0.56 – 24.20 | 0.900 |
|  |  |  |  |
| **Interaction with age (ref: 18-39) when living with children** |  |  |  |
| 40-64 years old | 0.35 | 0.15 – 0.82 | 0.016 |
| 65+ years old | 0.21 | 0.01 – 2.95 | 0.253 |
|  |  |  |  |
| Observations* | 413/442 |  |  |
| R2 Nagelkerke | 0.195 |  |  |

* Only analyzed in Participants who stated overall difficulty

**Table S5b** describes association of sociodemographic background with the perceived difficulty level of adherence to isolation recommendations overall in all 413 participants who stated their overall difficulty level in the retrospective cohort.

# Table S6

| **Table S6: Reasons for PCR testing** | | | |
| --- | --- | --- | --- |
| Total Cohort | | | |
|  | **prospective** | **retrospective** | **Total** |
|  | (N=1105) | (N=442) | (N=1547) |
| **Reason for PCR Testing** |  |  |  |
| Symptoms | 661 (59.8%) | 315 (71.3%) | 976 (63.1%) |
| Contacted by direct contact | 287 (26.0%) | 65 (14.7%) | 352 (22.8%) |
| Contacted by officials | 55 (5.0%) | 11 (2.5%) | 66 (4.3%) |
| Travel | 23 (2.1%) | 3 (0.7%) | 26 (1.7%) |
| Hospital/GP other reason | 19 (1.7%) | 10 (2.3%) | 29 (1.9%) |
| Precaution | 18 (1.6%) | 8 (1.8%) | 26 (1.7%) |
| Employer/School/Military | 16 (1.4%) | 18 (4.1%) | 34 (2.2%) |
| SwissCovid-App* | 11 (1.0%) | 0 (0%) | 11 (0.7%) |
| Other | 5 (0.5%) | 3 (0.7%) | 8 (0.5%) |
| Missing | 10 (0.9%) | 9 (2.0%) | 19 (1.2%) |

*Not available until June 25th, 2020

# Tables S7a – S7c

| **Table S7a: Compliance after confirmed SARS-CoV-2 Infection** | | | | | | | | | | |
| --- | --- | --- | --- | --- | --- | --- | --- | --- | --- | --- |
| Prospectively recruited Cohort | | | | | | | | | | |
|  | **18-39 years** | | | **40-64 years** | **65+ years** | | | **Total** | | |
| **Adhere to Isolation** | **(N=344)** | | | **(N=448)** | **(N=313)** | | | **(N=1105)** | | |
| Always | 263 (76.5%) | | | 332 (74.1%) | 205 (65.5%) | | | 800 (72.4%) | | |
| Almost always | 31 (9.0%) | | | 49 (10.9%) | 21 (6.7%) | | | 101 (9.1%) | | |
| Frequently | 3 (0.9%) | | | 4 (0.9%) | 4 (1.3%) | | | 11 (1.0%) | | |
| Occasionally | 1 (0.3%) | | | 1 (0.2%) | 3 (1.0%) | | | 5 (0.5%) | | |
| Rarely | 2 (0.6%) | | | 3 (0.7%) | 0 (0%) | | | 5 (0.5%) | | |
| Very rarely | 3 (0.9%) | | | 4 (0.9%) | 2 (0.6%) | | | 9 (0.8%) | | |
| Never | 3 (0.9%) | | | 3 (0.7%) | 2 (0.6%) | | | 8 (0.7%) | | |
| Missing | 38 (11.0%) | | | 52 (11.6%) | 76 (24.3%) | | | 166 (15.0%) | | |
| **Table S7b: Compliance before confirmed SARS-CoV-2 Infection** | | | | | | | | | | |
| Prospectively recruited Cohort | | | | | | | | | | |
| When Reason for Testing was having symptoms | | | | | | | | | | |
|  | | **18-39 years** | **40-64 years** | | | **65+ years** | | | | **Total** |
| **Adhere to Quarantine** | | **(N=204)** | **(N=270)** | | | **(N=187)** | | | | **(N=661)*** |
| Always | | 114 (55.9%) | 158 (58.5%) | | | 94 (50.3%) | | | | 366 (55.4%) |
| Almost always | | 35 (17.2%) | 55 (20.4%) | | | 41 (21.9%) | | | | 131 (19.8%) |
| Frequently | | 10 (4.9%) | 16 (5.9%) | | | 9 (4.8%) | | | | 35 (5.3%) |
| Occasionally | | 20 (9.8%) | 2 (0.7%) | | | 7 (3.7%) | | | | 29 (4.4%) |
| Rarely | | 6 (2.9%) | 5 (1.9%) | | | 1 (0.5%) | | | | 12 (1.8%) |
| Very rarely | | 9 (4.4%) | 2 (0.7%) | | | 2 (1.1%) | | | | 13 (2.0%) |
| Never | | 3 (1.5%) | 5 (1.9%) | | | 5 (2.7%) | | | | 13 (2.0%) |
| Missing | | 7 (3.4%) | 27 (10.0%) | | | 28 (15.0%) | | | | 62 (9.4%) |
| **Table S7c: Compliance before confirmed SARS-CoV-2 Infection** | | | | | | | | | | |
| Prospectively recruited Cohort | | | | | | | | | | |
| When Reason for Testing was not connected to having symptoms | | | | | | | | | | |
|  | | **18-39 years** | **40-64 years** | | | | **65+ years** | | **Total** | |
| **Adhere to Quarantine** | | **(N=139)** | **(N=174)** | | | | **(N=121)** | | **(N=434)*** | |
| Always | | 88 (63.3%) | 115 (66.1%) | | | | 85 (70.2%) | | 288 (66.4%) | |
| Almost always | | 22 (15.8%) | 28 (16.1%) | | | | 19 (15.7%) | | 69 (15.9%) | |
| Frequently | | 7 (5.0%) | 3 (1.7%) | | | | 5 (4.1%) | | 15 (3.5%) | |
| Occasionally | | 7 (5.0%) | 2 (1.1%) | | | | 0 (0%) | | 9 (2.1%) | |
| Rarely | | 3 (2.2%) | 1 (0.6%) | | | | 0 (0%) | | 4 (0.9%) | |
| Very rarely | | 1 (0.7%) | 2 (1.1%) | | | | 0 (0%) | | 3 (0.7%) | |
| Never | | 3 (2.2%) | 8 (4.6%) | | | | 5 (4.1%) | | 16 (3.7%) | |
| Missing | | 8 (5.8%) | 15 (8.6%) | | | | 7 (5.8%) | | 30 (6.9%) | |

# Figure S1a

Flowchart for the prospectively recruited population sample, detailing the process of recruitment from all 72394 individuals reported to contact tracing between August 6^th^, 2020, and January 26^th^, 2021.

# Figure S1b

Flowchart for the retrospectively recruited population sample, detailing the process of recruitment from all 72394 individuals reported to contact tracing between February 27^th^, 2020, and August 5^th^, 2020.

# Figure S2


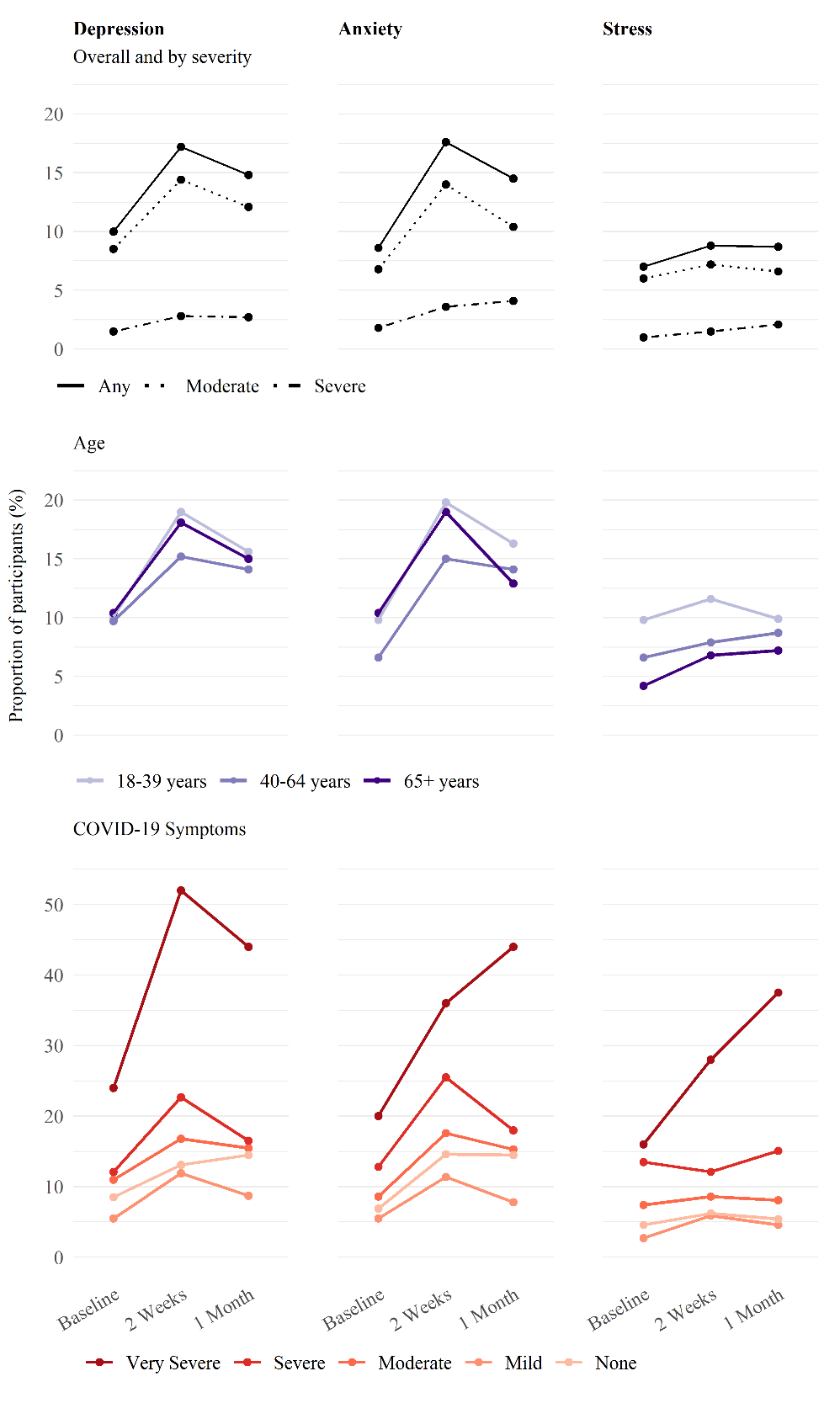


**Figure S2: Mental Burden of isolation – Sensitivity Analysis,** visualizes the percentage of the population reporting symptoms of depression, anxiety, or stress in those participants who filled out their DASS-21 questionnaire at all 3 timepoints, baseline, 2-weeks, and 1-month after confirmed infection (complete data only, n=931).

# Figures S3a – S3c


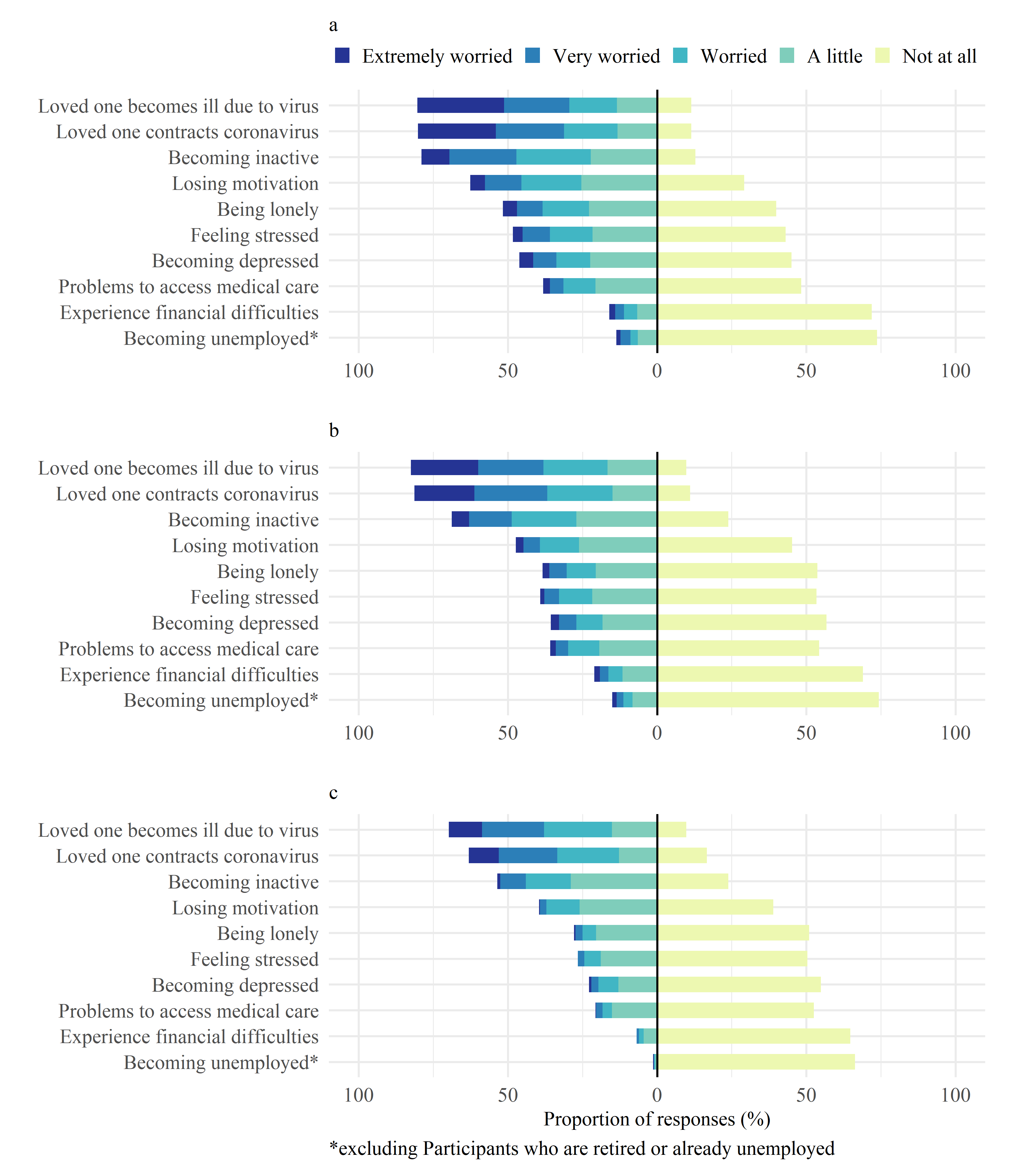


**Figures S3a to S3c.** Worries of participants during isolation, subgroup analysis in the age groups, (a) 18-39 years old, (b) 40-64 years old, (c) over 65 years old

# Figures 4a – 4c


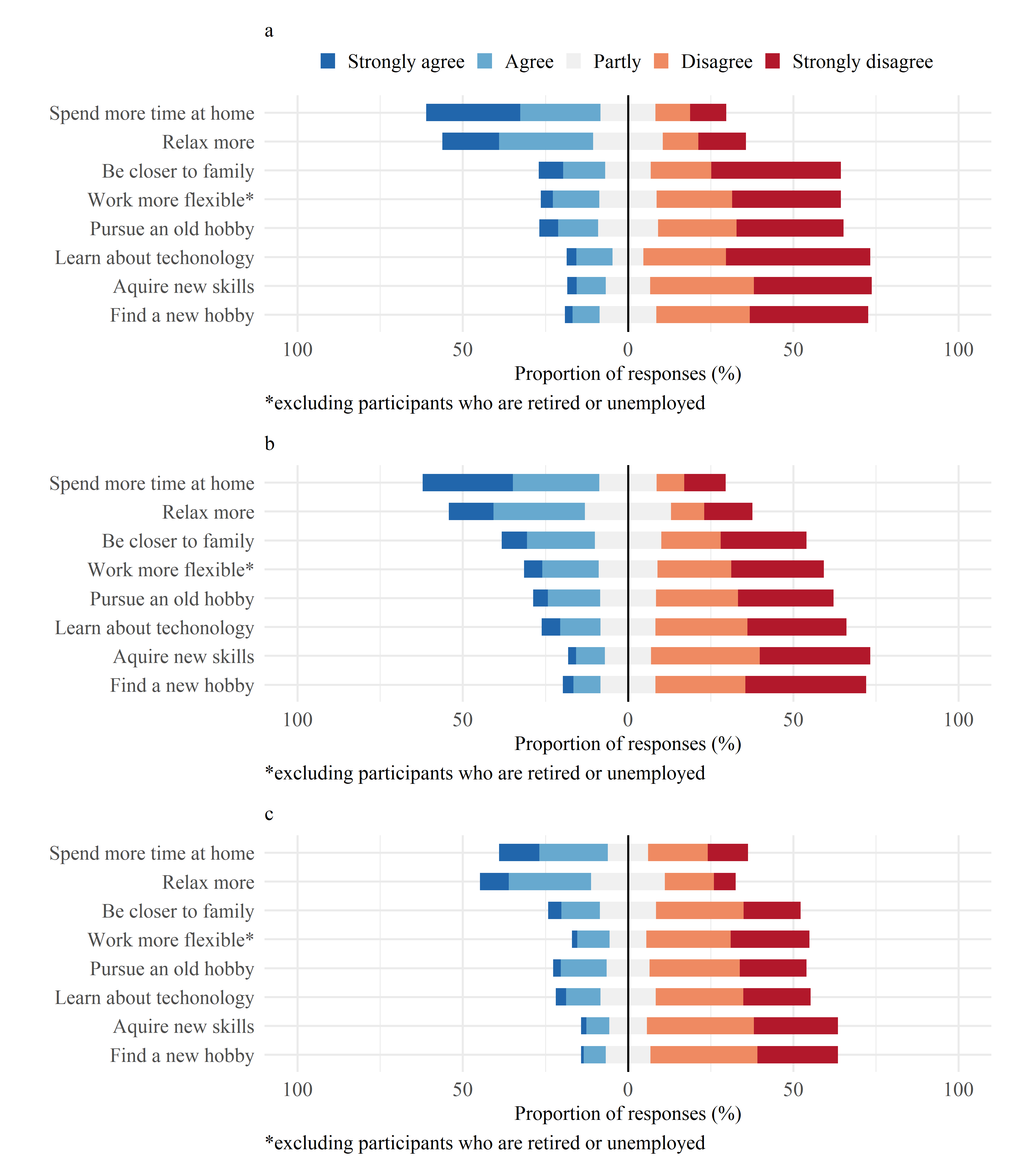


**Figures S4a to S4c.** Positive aspects of isolation, subgroup analysis in the age groups, (a) 18-39 years old, (b) 40-64 years old, (c) over 65 years old

# Figures S5a – S5c


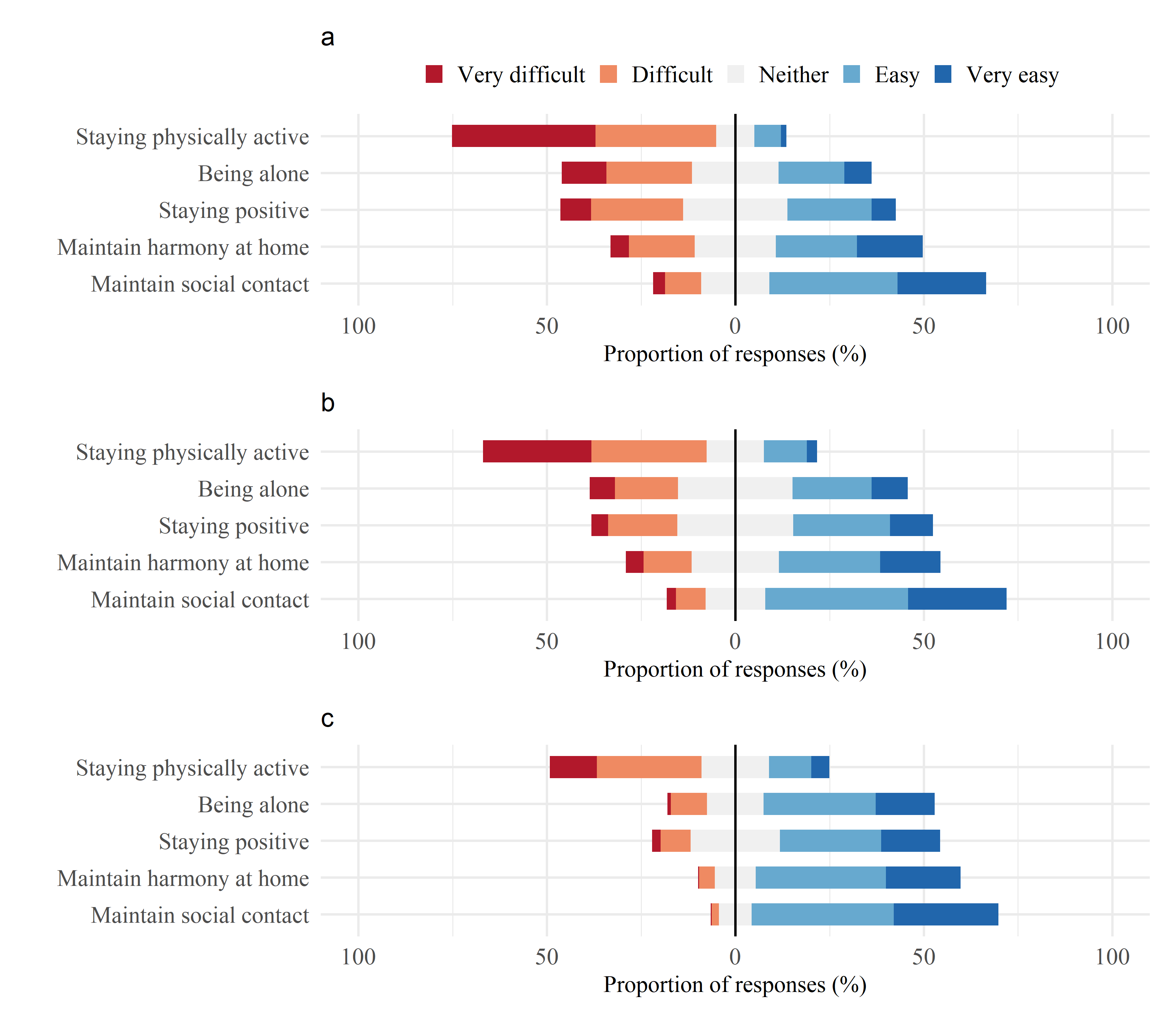


**Figures S5a to S5c.** Difficulties of isolation, subgroup analysis in the age groups, (a) 18-39 years old, (b) 40-64 years old, (c) over 65 years old
