# Supplementary material for: Mental health of individuals infected with SARS-CoV-2 during mandated isolation and compliance with recommendations—A population-based cohort study
Source: PLoS One. 2022 Mar 16;17(3):e0264655. doi: 10.1371/journal.pone.0264655 (PMC8926272; doi:10.1371/journal.pone.0264655)
Supplement: Attachment — Submitted filename: renamed_ac8e8.docx [file pone.0264655.s003.docx]

**COMMENTS – Manuscript PONE-D-21-39431**

Title: “Mental Health of Individuals Infected with SARS-CoV-2 during Mandated Isolation and Compliance with Recommendations - a Population-Based Cohort Study”

**Important note: This review pertains only to ‘statistical aspects’ of the study and so ‘clinical aspects’ [like medical importance, relevance of the study, ‘clinical significance and implication(s)’ of the whole study, etc.] are to be evaluated [should be assessed] separately/independently. Further please note that any ‘statistical review’ is generally done under the assumption that (such) study specific methodological [as well as execution] issues are perfectly taken care of by the investigator(s). This review is not an exception to that and so does not cover clinical aspects {however, seldom comments are made only if those issues are intimately / scientifically related & intermingle with ‘statistical aspects’ of the study}. Agreed that ‘statistical methods’ are used as just tools here, however, they are vital part of methodology [and so should be given due importance]. To improve the article/presentation, clues/hints may be taken from this review but should not limit the process by adhering to those points alone.**

**COMMENTS: Although the study is very important, of current interest and the study is well conducted, I have few concerns. According to account given in lines 116-122 [**We enrolled two populations of SARS-CoV-2 infected individuals. The first, henceforth referred to as “retrospectively recruited”, included all eligible individuals who were diagnosed with a SARS-CoV-2 infection prior to the start of the study (i.e., between 27 February 2020 and 05 August 2020). These individuals were enrolled between 06 October 2020 and 26 January 2021, at a median of 7.2 months after their diagnosis. The second, referred to as “prospectively recruited”, included an age-stratified random sample of all eligible individuals diagnosed between 06 August 2020 and 19 January 2021. These individuals were enrolled upon or shortly after diagnosis.**], however, for analyses (example: application of ‘multivariable ordinal regression’ – table-2) you seem to have combined these samples. Even if (refer to lines 203-4) observing that “There were no considerable differences regarding the sociodemographic characteristics of the two sample populations (Table 1)”, is that {combining samples} correct? [Agreed that there were no considerable differences regarding the sociodemographic characteristics but the fact is these two samples collected by different methods]**

**In ‘abstract-** **Methods and Findings’ section, lines 33-35 only prospective cohort study [“This population-based prospective cohort study enrolled 1547 adults from the general population with SARS-CoV-2 infection] is mentioned. This mixing confusion is seen at other places also {example: Section on ‘Study design and Population’ (line 108 onwards) “This analysis is based on the Zurich SARS-CoV-2 Cohort study, an ongoing observational, population-based cohort study of individuals in the Canton of Zurich with polymerase chain reaction (PCR) -confirmed SARS-CoV-2 infection”. Please address this issue to clarify more. [No problem if it is so, but should clarified,** remember that this is a scientific/academic document and so all details should be clearly/correctly communicated**]**

**In the context of information given in lines 144-46 [“To evaluate worries, positive experiences, and difficulties during isolation, we used five-point Likert scale questions with scales ranging from “extremely worried” to “not worried at all”, “strongly agree” to “strongly disagree” and “very difficult” to “very easy”, respectively], please note the following {** **pasted from one standard textbook on ‘Research Methodology’}:**

Whenever response options ranged from 1=strongly disagree to 4=strongly agree (or ranging from 1 (strongly disagree) to 6 (strongly agree) or from 1=very bad to 3=neither good nor bad to 5=very good), while using a ‘Likert’ scale responses, recoding [like strongly disagree=-2, disagree=-1, neutral=0, agree=1, strongly agree=2] may yield correct and meaningful ‘arithmetic mean’ which is useful not only for comparison but has absolute meaning. Application of any statistical test(s) assume that meaning of entity used (mean, SD, etc) has a particular meaning. Though ‘α’ [alpha] or most other measures of reliability/correlation will remain same, however, use of non-parametric methods should/may be preferred while dealing with data yielded by any questionnaire/score.

**Also consider that reported (observed) value of Nagelkerke’s R2 (=0.127) in table-2 is small and table-3 ( R2=0.068 ) is very very small. That the amount of variation explained is all most negligible. From (lines 157-8:** Calculation and imputation of DASS-21 scores is described in previously published research from our study and followed official guidance (21).**) it seems that the present sample is a sub-sample of some other study. If so, why this fact is not made clear in the beginning? Again, remember that this is a scientific/academic document and so all details should be clearly/correctly communicated.**

**Most of the statistical values/results are not interpreted adequately [example: Odds Ratios in table-3, male Sex (=0.90); Living Alone (=0.60)]. Study has potential, however, I suggest to consider above points.**
